# Genotypic and phenotypic characteristics of Turkish patients with phenylalanine metabolism disorders

**DOI:** 10.1007/s11011-025-01582-1

**Published:** 2025-04-28

**Authors:** Fatma-Nur Kuzucu, Mustafa Kilic, Abdullah Sezer, Selen Has-Ozhan, Harun Yildiz, Tuba Celen-Yoldas, Fatma-Nese Onat, Melike Uyanik

**Affiliations:** 1 Department of Pediatrics, Sami Ulus Children Hospital, Ankara, Turkey; 2 Department of Pediatrics, University of Health Sciences, Sami Ulus Children Hospital, Metabolism Unit, Babur Cad. No: 44, 06080 Altındag Ankara, Turkey; 3 Department of Medical Genetics, Sami Ulus Children Hospital, Ankara, Turkey; 4 Department of Developmental Pediatrics, Sami Ulus Children Hospital, Ankara, Turkey; 5 Department of Dietetics and Nutrition, Sami Ulus Children Hospital, Ankara, Turkey

**Keywords:** Hyperphenylalaninemia, Phenylketonuria, Tetrahydrobiopterin, Phenylalanine, Newborn screening, PAH

## Abstract

Phenylketonuria (PKU) is an autosomal recessive disorder of phenylalanine metabolism, in which especially high phenylalanine concentrations cause brain dysfunction. If untreated, this brain dysfunction results in severe intellectual disability, epilepsy, and behavioural problems. We aimed to investigate demographic, clinical, biochemical, and molecular genetic data in patients with phenylalanine metabolism disorder. This study included 99 predominantly Turkish patients diagnosed with phenylalanine metabolism disorder, primarily referred through newborn screening programs. These patients were evaluated at a single center over a 9-year period, from 2013 to 2021. Demographic, clinical, molecular and laboratory data were collected retrospectively. Among the 99 patients, 93 (93.9%) had hyperphenylalaninemia-phenylketonuria, 2 (2.0%) had tetrahydrobiopterin metabolism disorders [one due to 6-pyruvoyl-tetrahydropterin synthase (PTPS) deficiency and the other due to dihydropteridine reductase (DHPR) deficiency], 3 (3.0%) had maternal PKU syndrome (one of whom also had mild phenylketonuria), and 1 (1.0%) had transient hyperphenylalaninemia. The majority of patients belonged to the mild hyperphenylalaninemia-not requiring treatment group. A total of 33 different alleles and 40 genotypes (59.6% compound heterozygous) were identified in the *PAH* gene, with missense variants accounting for the largest proportion (72.7%). The most frequent *PAH* gene variants were c.898G > T p.(Ala300Ser) (14.9%), c.1066-11G > A (8.5%), and c.1208C > T p.(Ala403Val) (8.5%), while the most common genotypes were c.898G > T p.(Ala300Ser)/c.898G > T p.(Ala300Ser) (6.4%) and c.898G > T p.(Ala300Ser) /c.1066-11G > A (6.4%), respectively. Among patients with mild hyperphenylalaninemia-not requiring treatment, the predominant genotypes were c.898G > T p.(Ala300Ser)/c.898G > T p.(Ala300Ser) (11.1%), c.898G > T p.(Ala300Ser)/c.1066-11G > A (11.1%), and c.1208C > T p.(Ala403Val)/c.1208C > T p.(Ala403Val) (7.4%), whereas c.842C > T p.(Pro281Leu)/c.842C > T p.(Pro281Leu) (33.3%) was frequently observed in classic PKU patients. The national newborn screening program has significantly improved the prognosis and quality of life for patients through early diagnosis and timely treatment. While the prevalence of hyperphenylalaninemia-phenylketonuria remains high in Turkey, the higher frequency of the hyperphenylalaninemia-not requiring treatment group, compared to European and Asian countries, is considered a favorable outcome. Additionally, the PAH genotype is identified as the primary determinant of the PKU phenotype.

## Introduction

Phenylketonuria (PKU, Følling disease, OMIM #261600), is an autosomal recessive disorder of phenylalanine (Phe) metabolism caused by biallelic pathogenic variants in the *PAH* gene encoding phenylalanine hydroxylase (PAH, EC 1.14.16.1, OMIM *612349). PAH is responsible for the conversion of Phe to tyrosine (Tyr), requiring the cofactor and/or chaperone tetrahydrobiopterin (BH4) and DnaJ homolog subfamily C member 12 (DNAJC12) with molecular oxygen and non-heme iron. Hyperphenylalaninemia (HPA) is most commonly caused by pathogenic variants in the *PAH* gene, but it is also caused by defects in BH4 metabolism or pathogenic variants in DNAJC12, which account for 1–3% of HPA. HPA is the core biochemical abnormality of PKU, in which normal blood Phe concentrations (35–120 μmol/l) are exceeded. Untreated patients develop severe intellectual disability, epilepsy, microcephaly, behavioural, psychiatric and movement problems, as well as light pigmentation of skin, eyes and hair, eczema and a musty odour (van Spronsen et al. 2021).

The prevalence of phenylketonuria varies worldwide, exhibiting significant ethnic and geographic differences. Globally, the average prevalence in newborns is approximately 1 in 24,000, while in Europe, it is about 1 in 10,000. The incidence is high (> 1:10,000) in Middle Eastern countries such as Turkey, Iran, and Jordan, as well as in North African countries like Egypt and certain European regions, including Italy and Northern Ireland. It is moderate (1:10,000–1:100,000) in countries such as the United States, Latin America, and China, and low (< 1:100,000) in regions like Finland, Thailand, Japan, and some parts of Africa (Hillert et al. 2020; Elhawary et al. 2022). Early diagnosis through newborn screening (NS) is crucial. If treatment begins promptly and is maintained, patients generally achieve intelligence within normal limits, though mild suboptimal neurocognitive outcomes persist. The cornerstone of treatment for the past 70 years has been dietary Phe restriction, which has proven highly effective, though adherence can be challenging and outcomes are not always ideal.

Pharmacological treatments are available, such as BH4 (sapropterin hydrochloride), which is effective in only a minority of patients and pegylated phenylalanine ammonia lyase (PAL, pegvaliase), which requires daily subcutaneous injections and causes adverse immune responses. BH4 enhances residual PAH activity and can lower Phe levels. BH4 responsiveness is defined as a ≥ 30% reduction in blood Phe levels within 24–48 h of administration or a two fold increase in dietary Phe tolerance following a 20 mg/kg dose (Blau et al. 2011, 2013). While most individuals with mild HPA or milder forms of PKU are responsive, only 10% of classic PKU patients respond to BH4. Common side effects include hypophenylalaninemia, headache, rhinorrhea, pharyngolaryngeal pain, vomiting, diarrhea, nasal congestion, and cough. Pegvaliase, an enzyme substitution therapy that degrades Phe and can lower Phe levels. Approved by the FDA in 2018 for adults in the USA and for patients ≥ 16 years of age in Europe with uncontrolled blood Phe levels (> 600 μmol/L). However, adverse events such as hypophenylalaninemia, skin reactions, arthralgia, and rare anaphylactic responses have been reported. Additional therapies include dietary supplementation with casein glycomacropeptide (GMP), which contains minimal aromatic amino acids and may enhance immune function, and large neutral amino acids (LNAA) to block Phe transport into the brain. Emerging treatments, such as mRNA-based and gene therapies, are under development and hold promise for addressing the underlying genetic defect (van Spronsen et al. 2021).

PKU was first described in two Norwegian siblings in 1934 by Følling, was first treated (with dietary control) by Bickel et al. in 1953. Population-based NS using dried blood spot (DBS) testing to assess Phe concentration was introduced in 1963 by Guthrie and Susi and this improvements enabled early diagnosis and initiation of treatment. Today, many countries around the world, including Turkey since 2006, include PKU in their national NS programmes (Ozalp et al. 2001; van Spronsen et al. 2021; Coşkun et al. 2022).

The *PAH* gene is located on chromosome 12q23.2 (Lidksy et al. 1984; Lidsky et al. 1985). The canonical transcript (NM_000277.3, ENST00000553106.6) of human *PAH* gene consists of 13 exons and 12 introns spanning approximately 90 Kb. Its mRNA encodes polypeptides of 452 amino acid residues which are comprised of three domains: an N-terminal regulatory domain (residues 1–142), a catalytic domain (residues 143–410) and a C-terminal domain (residues 411–452). PAH polypeptides assemble into a homotetrameric enzyme. More than 3000 variants of the *PAH* gene have been reported in the Phenylalanine Hydroxylase Gene Locus-Specific Database (*PAH*vdb), with over 1000 of them classified as pathogenic. Most of these variants are observed in the central catalytic domain and compound heterozygous for two different *PAH* variants. The majority of *PAH* variants are in-frame/missense amino acid substitutions (58.3%), whereas frameshift variants (13.9%), splice variants (13.1%), nonsense variants (6.9%) and synonymous substitutions (4.9%) are less common. Of pathogenetic variants, 17.9% occur in intronic or untranslated regions of the *PAH* gene. The underlying genetic heterogeneity is the basis of the biochemical and clinical diversity of this disorder. Genotype and phenotype correlations are important to guide treatment for PKU patients and they can predict the prognosis in majority of cases. Allelic interaction can complicate the phenotype prediction. In compound heterozygosity, the allele causing a milder phenotype is always dominant to the allele causing severe phenotypes (Hillert et al. 2020). There are obvious racial and regional differences, for example, p.(Arg408Trp) is the most common mutation in Eastern Europe and c.1066-11G > A is the most common in Middle East regions such as Iran and Turkey, as well as Southern Europe (Hillert et al. 2020; Van Spronsen et al. 2021).

This study retrospectively analyzes the demographic, clinical, biochemical, and molecular genetic data of HPA/PKU patients to elucidate genotype–phenotype correlations, with a particular focus on individuals of predominantly Turkish descent.

## Materials and methods

### Patient registration

This study included all patients diagnosed with Phe metabolism disorders, either by clinical and biochemical analyses, with or without molecular genetic testing, during a 9-year period from 2013 to 2021 at our single-center. Demographic, clinical, and laboratory data, including plasma Phe levels and/or molecular genetic results, were retrospectively collected. Clinical and demographic information, as well as the mode of HPA/PKU diagnosis—either early detection through NS or late clinical diagnosis—were documented. The diagnosis of HPA/PKU is suspected when blood Phe levels exceed 2 mg/dL (> 120 μmol/L) in a NS sample. Newborns with persistently elevated Phe levels (> 2 mg/dL) and a Phe/Tyr ratio > 1.5 are referred to treatment centers for further evaluation and management. Plasma Phe levels were measured by ion-exchange chromatography using a single-column amino acid analyzer (Beckman 6300, Beckman Instruments, Palo Alto, California, USA). Plasma samples were collected, and patients with blood Phe levels above the normal threshold (normal ≤ 2 mg/dL or ≤ 120 μmol/L) were included in the study. Patients were classified into biochemical phenotypes based on the highest untreated blood Phe concentration, as follows: Classic (severe) PKU: blood Phe > 1200 μmol/L or > 20 mg/dL, Moderate PKU: blood Phe 900–1200 μmol/L or 15–20 mg/dL, Mild PKU: blood Phe 600–900 μmol/L or 10–15 mg/dL, Mild HPA-gray zone: blood Phe 360–600 μmol/L or 6–10 mg/dL, Mild HPA-not requiring treatment: blood Phe 120–360 μmol/L or 2–6 mg/dL (Camp et al. 2014). Dietary treatment (Phe restriction) and/or BH4 therapy are implemented if blood Phe levels are ≥ 6 mg/dL (≥ 360 μmol/L).

According to the European guidelines of 2017, good dietary compliance was defined as a median blood Phe level of 2–6 mg/dL in children under 12 years of age, and 2–10 mg/dL in children aged 12 years and older. Blood Phe levels above these thresholds were considered indicative of poor dietary compliance during follow-up (Van Spronsen et al. 2017).

Standardized growth curves for Turkish children were used to assess the anthropometric measurements of the patients, and standard deviation scores (SDS) were calculated. Developmental evaluation was conducted in the developmental pediatrics outpatient clinic of our hospital using the Early Development Inventory (EGE), Bayley Scales, and the Developmental Monitoring and Support Guide (GIDR).

### Genetic analyses

Molecular analysis of the *PAH, PTS* and *QDPR* genes were performed on genomic DNA by next generation sequencing (NGS) methods using DNA extracted from peripheral lymphocytes. Multiplex ligation probe amplification (MLPA) was performed on one patient because of only a single heterozygous mutation was detected by NGS or Sanger sequencing. For the reference sequence of *PAH*, *PTS* and *QDPR* genes, NM_000277.3, NM_000317.3 and NM_000320.3 transcipts were used, respectively.

The majority of patients were assessed by NGS using proprietary primers and sequenced using Miseq Illumina equipment (Illumina, San Diego, CA, USA). In a limited number of patients, Sanger sequencing was used to sequence the entire *PAH* gene. Sanger sequencing was mostly used to study family members. Only one patient suspicious for copy number variation was evaluated by MLPA by using SALSA MLPA P209-C2 kit (MRC-Holland, Amsterdam, the Netherlands). Sequence variants were interpreted according to the recommendations of the American College of Medical Genetics and Genomics and the Association for Molecular Pathology (Richards et al. 2015).

In silico predictions for pathogenicity in missense variants was evaluated using the REVEL (Ioannidis et al. 2016), BayesDel2 (Feng 2017) and FATHMM (http://fathmm.biocompute.org.uk/fathmm-xf/) tools. Splicing predictions were assessed by SpliceAI tool (https://spliceailookup.broadinstitute.org/). The GnomAD (https://gnomad.broadinstitute.org/) and Turkish Variome (Kars et al. 2021) datasets were used to obtain variant frequencies in control populations. The ClinVar (https://www.ncbi.nlm.nih.gov/clinvar/), HGMD (http://www.hgmd.cf.ac.uk/ac/index.php) and LOVD (https://www.lovd.nl/) databases were used to access clinically classified variants.

### Statistical analysis

Statistical evaluation was done using Statistical Package for Social Sciences (SPSS) for Windows 20 (IBM SPSS Inc., Chicago, IL). The normal distribution of the data was evaluated with the Kolmogorov–Smirnov test. Numerical variables without normal distribution were presented as median (min–max). Categorical variables were specified as numbers and percentages. Student T test was used to compare numerical variables that showed normal distribution between the groups with disease-onset before or after 2 weeks of life, and Mann–Whitney U test was used to compare numerical variables that did not show normal distribution. In statistical analysis, p < 0.05 was considered significant.

### Ethical approval

The study protocol was approved by the Local Medical Research Ethics Committee of Sami Ulus Training and Research Hospital with the number of 2021/06-183 and conducted in accordance with the Declaration of Helsinki.

## Results

A total of 99 patients were enrolled in this study. Of these, 94 (94.9%) were of Turkish descent, while 3 (3.0%) were Syrian and 2 (2.0%) were Iraqi. The cohort included 56 females (56.6%) and 43 males (43.4%). Most patients resided in the Central Anatolian region, primarily in the capital city, Ankara. Parental consanguinity was absent in 64 patients (64.6%), while it was observed in 35.4% of cases: 20 patients (20.2%) had parents who were first-degree cousins, 6 (6.1%) had second-degree cousin parents, and 9 (9.1%) had third-degree cousin parents. A family history of a similar disorder was reported in 13 patients (13.3%). Importantly, 91 patients (91.9%) were diagnosed early through the NS program, including one patient with maternal PKU syndrome and mild PKU. Two patients (2.0%) were evaluated due to maternal PKU syndrome, while the remaining 6 patients (6.1%) were diagnosed late based on clinical presentation. Of note, all but six patients (five of these were foreign nationals), participated in the NS program.

The phenotypes of the patients were categorized as follows: Out of 99 patients, 74 (75%) had mild HPA, with 55 (74.3%) classified as mild HPA-not requiring treatment and 19 (25.7%) in the mild HPA-gray zone. Additionally, 4 patients (4%) had mild PKU, 3 (3%) moderate PKU, and 12 (12%) classic PKU. Rare phenotypes included 1 patient (1%) with DHPR deficiency, 1 (1%) with PTPS deficiency, 3 (3%) with maternal PKU syndrome (one of whom also had mild PKU), and 1 patient (1%) with transient HPA.

The median current age of the patients was 33 months (range: 3–186 months). A total of 77 patients (78%) were in the neonatal period (0–30 days), 15 patients (15%) were in the infant period (1–24 months), 3 patients (3%) were in childhood (2–10 years), and 1 patient (1%) was in the adolescent period (10–19 years) at the time of hospital admission. The age at admission for 3 patients (3%) was unknown, and no adult patients were included in the study. The median age at presentation was 20 days, while the median age of diagnosis was 26 days. Symptoms or signs were present at the time of admission in 6 patients (6%), with the median age of symptom onset being 180 days. A statistically significant moderate positive correlation was observed between the delay in presentation and the increase in blood Phe levels at the time of diagnosis (*p* < 0.05).

A history of comorbid diseases, either related or unrelated to HPA/PKU, was reported in 23 patients (23.2%). These included atopic dermatitis, cow's milk allergy, hemangioma, microcephaly, neurodevelopmental disorders (such as intellectual disability, hyperactivity, autism), congenital heart disease, and hydronephrosis. Additionally, some patients were diagnosed with rare diseases, including Niemann-Pick type C (NPC), achondroplasia, and Down syndrome. A history of seizures was present in 3 patients (3%). Microcephaly was observed in 12 patients (12.6%), including 5 with classic PKU, 3 with maternal PKU syndrome, 1 with moderate PKU, 1 with mild PKU, 1 with mild HPA, and 1 with PTPS deficiency. Malnutrition was found in 9 patients (9.2%), including 4 with classic PKU, 2 with maternal PKU syndrome, 1 with moderate PKU, 1 with mild PKU, and 1 with PTPS deficiency. Short stature was noted in 9 patients (9.2%), including 5 with classic PKU, 1 with maternal PKU syndrome, 1 with moderate PKU, 1 with mild HPA, and 1 with PTPS deficiency.

The median blood Phe levels of the patients included in the study were monitored during follow-up. It was observed that 9 patients (9.1%) had blood Phe levels above 6 mg/dl, while 90 patients (90.9%) had levels between 2–6 mg/dl. A total of 59 patients (59.6%) were followed without treatment, 35 patients (35.4%) were on dietary therapy only, 2 patients (2%) received both diet and BH4 therapy, 1 patient (1%) was treated with only BH4, 1 patient (1%) received BH4, L-dopa, and serotonin (due to PTPS deficiency), and 1 patient (1%) was treated with L-dopa, serotonin, and folinic acid (due to DHPR deficiency) in addition to dietary therapy. Among the 74 patients with mild HPA, 55 (74.3%) were followed without treatment, 17 patients (23%) received dietary therapy, 1 patient (1.4%) received BH4 therapy, and 1 patient (1.4%) was on both diet and BH4 therapy.

The median blood Phe levels of the patients receiving diet therapy and their compliance with treatment were evaluated. Among the 39 patients who received dietary therapy, the median blood Phe level of 30 patients (76.9%) was between 2–6 mg/dl, and their dietary compliance was considered good. The median blood Phe level of 9 patients (23.1%) was above 6 mg/dl, and their dietary compliance was poor. Of the 9 patients with poor dietary adherence, 4 (44.4%) had classic PKU, 3 (33.3%) had mild HPA-gray zone, 1 (11.1%) had moderate PKU, and 1 (11.1%) had mild PKU. A comparison of male and female patients in terms of dietary adherence revealed no statistically significant difference.

**Fig. 1:**
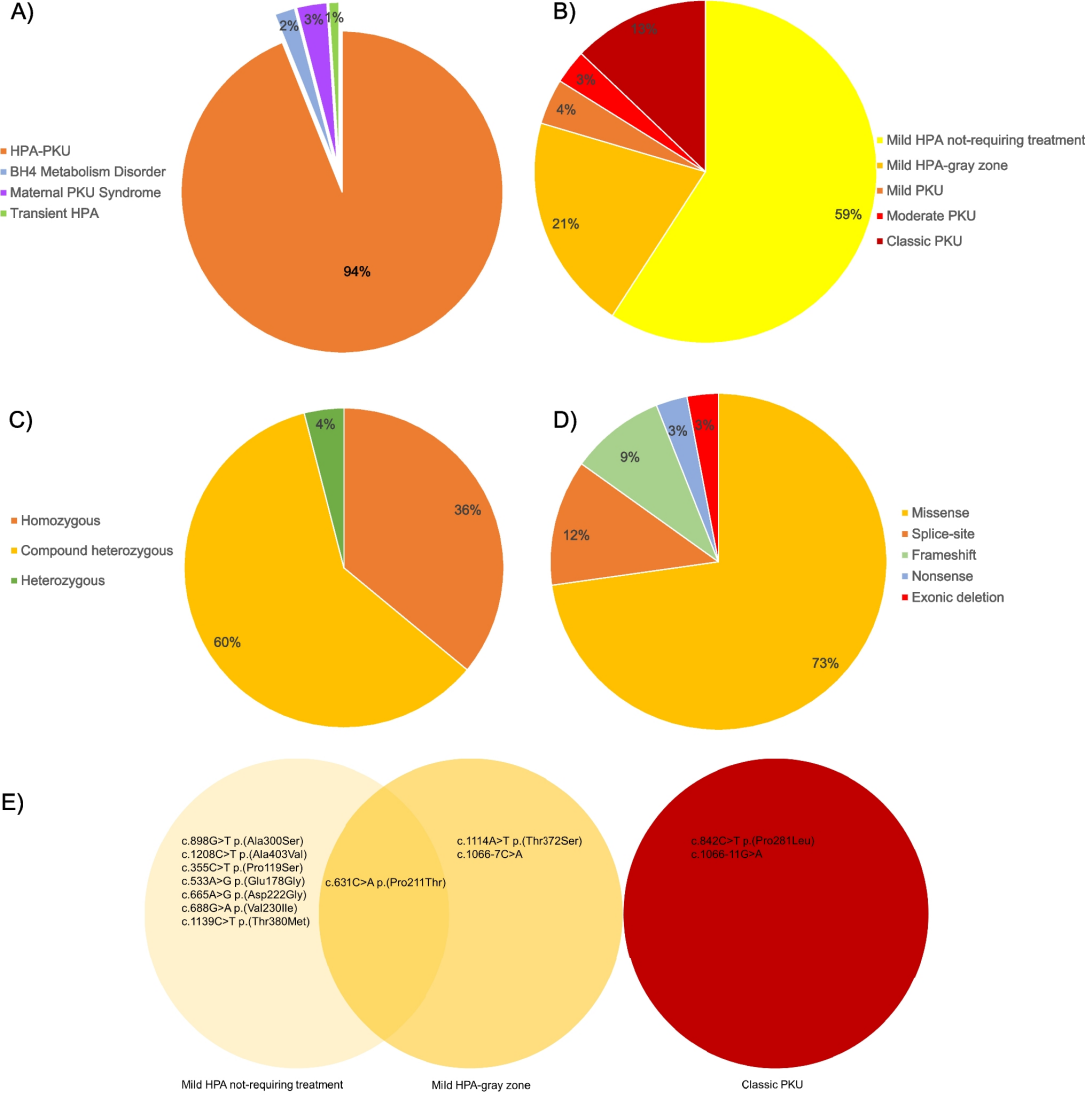
Visual diagrams of patient and variant subgroups. **A**. Classification of patients diagnosed with phenylalanine metabolism disorders (*n* = 99). **B.** Classification of patients diagnosed with hyperphenylalaninemia-phenylketonuria (HPA-PKU) (*n* = 94). **C.** Distribution of zygosity for *PAH* pathogenic variants identified in patients diagnosed with HPA-PKU (*n* = 47). **D.** Distribution of *PAH* pathogenic variants in HPA-PKU patients by variant type (*n* = 47). **E.** Clinical subgroups of *PAH* gene pathogenic variants observed in a homozygous state

**Fig. 2:**
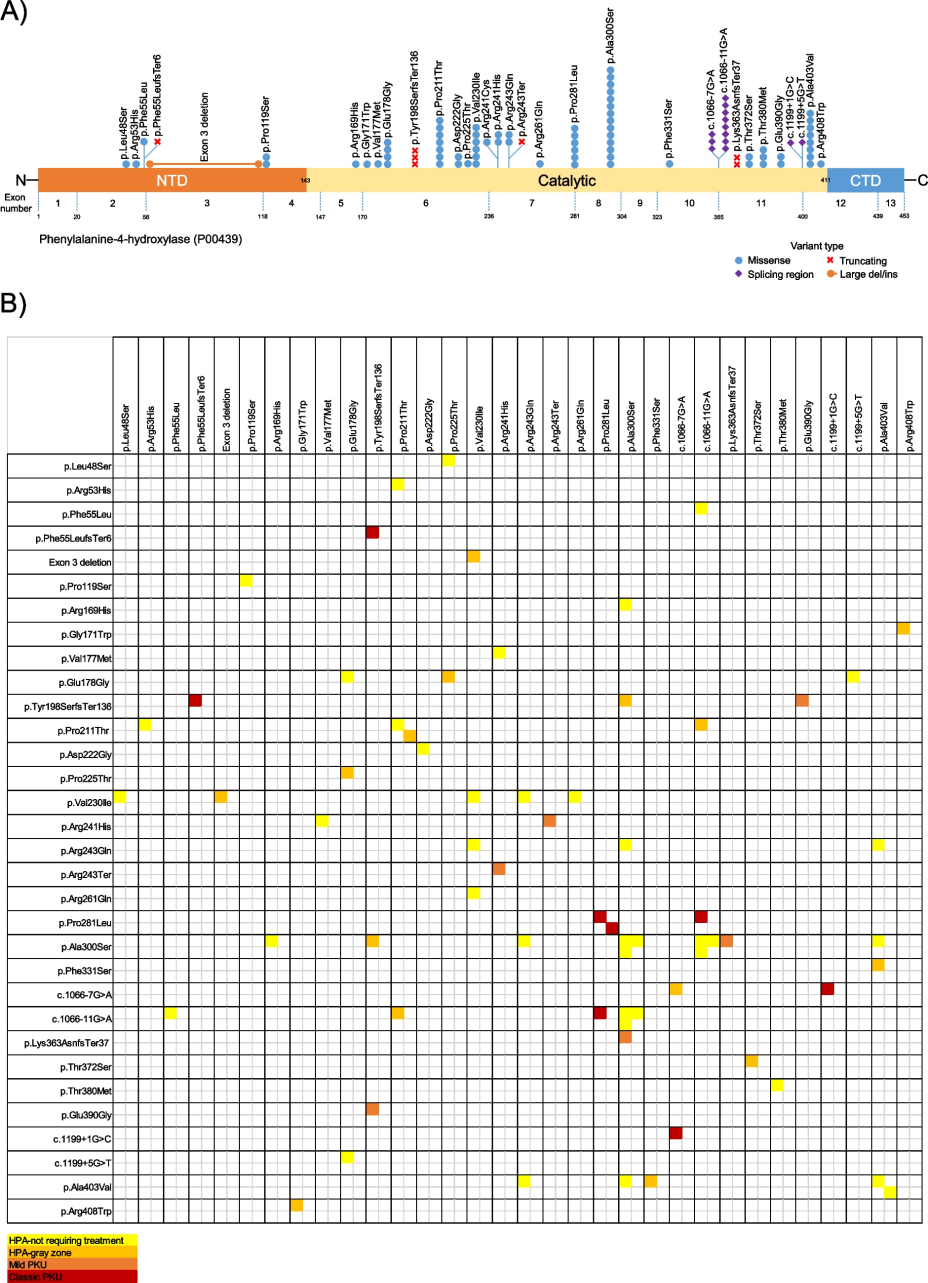
Genotypic and phenotypic characteristics of the *PAH* variants detected in the study cohort. **A.** Schematic representation of the PAH protein, illustrating the distribution of pathogenic variants. The number of beads represents the occurrence frequency of each variant within the cohort. NTD: N-terminal domain; CTD: C-terminal domain. **B.** Genotype–phenotype correlations of PAH variants. Each column and row represents one allele. Framed squares indicate biallelic states formed by variants corresponding to each column and row. Colored squares represent individual patients within the study cohort, highlighting their specific genotype and phenotype relationships

The distribution of genetic variants among HPA/PKU patients is presented in Tables 1, 2, 3, and 4, and Figs. 1 and 2. Molecular genetic analysis was performed on 50 patients, of whom 49 had pathogenic variants. Among these, 28 patients (59.6%) had genotypes in a compound heterozygous state, 17 patients (36.2%) had genotypes in a homozygous state, and 2 patients (4.2%) had genotypes in a single heterozygous state in the *PAH* gene. Additionally, one patient had a homozygous *PTS* genotype, and another had a homozygous *QDPR* genotype. *PAH* gene analysis was normal in one patient who was diagnosed with transient HPA during follow-up. Among the detected variants in the *PAH* gene, 24 (72.7%) were missense, 4 (12.1%) were splice-site variants, 3 (9.1%) were frameshift variants, 1 (3%) was an intragenic single-exon deletion, and 1 (3%) was a nonsense variant. In total, 35 different alleles (33 in the *PAH* gene, 1 in the *PTS* gene, and 1 in the *QDPR* gene) and 42 different genotypes (40 in the *PAH* gene, 1 in the *PTS* gene, 1 in the *QDPR* gene) were identified. Except for the novel variant detected in the *PTS* gene, all variants had been previously reported. Among the 33 alleles in the *PAH* gene, the most common variants were c.898G > T p.(Ala300Ser) (14.9%), c.1066-11G > A (8.5%), c.1208C > T p.(Ala403Val) (8.5%), c.631C > A p.(Pro211Thr) (7.5%), c.688G > A p.(Val230Ile) (6.4%), c.842C > T p.(Pro281Leu) (6.4%), and c.533A > G p.(Glu178Gly) (4.3%). The most common genotypes were c.898G > T p.(Ala300Ser)/c.898G > T p.(Ala300Ser) (6.4%) and c.898G > T p.(Ala300Ser)/c.1066-11G > A (6.4%).

**Table 1:** Distribution of *PAH* gene variants in HPA/PKU patients (n: 47)

Nucleotide change	Amino acid change	dbSNP	Variant type	ClinVar classification	Gene region Exon (E)/Intron /I)	Protein domain/Enzyme activity (%)	APV	Phenotype	Number of alleles/allele frequency (%)
Alleles (*n* = 94)									
c.898G > T	p.(Ala300Ser)	rs5030853	Missense	Pathogenic	E8	C/65	9.2	Mild HPA	14 (14.9)
c.1066-11G > A	p.?	rs5030855	Splice site	Pathogenic	I10	?/5	0	Classic PKU	8 (8.5)
c.1208C > T	p.(Ala403Val)	rs5030857	Missense	Pathogenic	E12	C/66	9.3	Mild HPA	8 (8.5)
c.631C > A	p.(Pro211Thr)	rs62514931	Missense	Pathogenic	E6	C/72	9.7	Mild HPA	7 (7.5)
c.688G > A	p.(Val230Ile)	rs62516152	Missense	Pathogenic	E6	C/66	9.8	Mild HPA	6 (6.4)
c.842C > T	p.(Pro281Leu)	rs5030851	Missense	Pathogenic	E7	C/2	0	Classic PKU	6 (6.4)
c.533A > G	p.(Glu178Gly)	rs77958223	Missense	Pathogenic	E6	C/39	7.4	Mild HPA- Mild PKU	4 (4.3)
c.592_613del	p.(Tyr198Serfs*136)	rs199475697	Frame shift	Pathogenic	E6	C/?	0	Classic PKU	3 (3.2)
c.1066-7C > A	p.?	rs1242756437	Splice site	Likely Pathogenic	I10	?/?	?	?	3 (3.2)
c.1139C > T	p.(Thr380Met)	rs62642937	Missense	Pathogenic	E11	C/28	9.9	Mild HPA	3 (3.2)
c.355C > T	p.(Pro119Ser)	rs398123292	Missense	Pathogenic	E4	R/?	6.7	Mild HPA- Mild PKU	2 (2.1)
c.665A > G	p.(Asp222Gly)	rs62507319	Missense	Pathogenic	E6	C/?	5.7	Mild PKU	2 (2.1)
c.722G > A	p.(Arg241His)	rs62508730	Missense	Pathogenic	E7	C/23	5.3	Mild PKU	2 (2.1)
c.728G > A	p.(Arg243Gln)	rs62508588	Missense	Pathogenic	E7	C/14	0	Classic PKU	2 (2.1)
c.1089delG	p.(Lys363Asnfs*37)	rs5030654	Frame shift	Pathogenic	E11	?/?	0	Classic PKU	2 (2.1)
c.1114A > T	p.(Thr372Ser)	rs62517163	Missense	Pathogenic	E11	C/?	8.5	Mild HPA	2 (2.1)
c.1169A > G	p.(Glu390Gly)	rs5030856	Missense	Pathogenic	E11	C/?	6.8	Mild HPA-Mild PKU	2 (2.1)
c.143 T > C	p.(Leu48Ser)	rs5030841	Missense	Pathogenic	E2	R/39	2.1	Mild PKU-Classic PKU	1 (1.1)
c.158G > A	p.(Arg53His)	rs118092776	Missense	VUS	E2	R/79	8.8	Mild HPA	1 (1.1)
c.165 T > G	p.(Phe55Leu)	rs199475598	Missense	Pathogenic	E2	R/?	8.3	Mild HPA	1 (1.1)
c.165delT	p.(Phe55Leufs*6)	rs199475566	Frame shift	Pathogenic	E2	?/?	0	Classic PKU	1 (1.1)
c.506G > A	p.(Arg169His)	rs199475679	Missense	Pathogenic	E5	C/52	10	Mild HPA	1 (1.1)
c.511G > T	p.(Gly171Trp)	rs199475613	Missense	Likely Pathogenic	E6	C/?	?	?	1 (1.1)
c.529G > A	p.(Val177Met)	rs199475602	Missense	Pathogenic	E6	?/?	10	Mild HPA	1 (1.1)
c.673C > A	p.(Pro225Thr)	rs199475589	Missense	Pathogenic	E6	C/?	0	Classic PKU	1 (1.1)
c.721C > T	p.(Arg241Cys)	rs76687508	Missense	Pathogenic	E7	C/?	6.0	Mild PKU	1 (1.1)
c.727C > T	p.(Arg243*)	rs5030846	Nonsense	Pathogenic	E7	C/?	0	Classic PKU	1 (1.1)
c.782G > A	p.(Arg261Gln)	rs5030849	Missense	Pathogenic	E7	C/44	1.7	Classic PKU	1 (1.1)
c.992 T > C	p.(Phe331Ser)	rs199475614	Missense	Pathogenic	E10	C/?	5.5	Mild PKU	1 (1.1)
c.1199 + 5G > T	p.?	rs62508674	Splice site	Likely Pathogenic	I11	?/?	0	Classic PKU	1 (1.1)
c.1222C > T	p.(Arg408Trp)	rs5030858	Missense	Pathogenic	E12	C/2	0	Classic PKU	1 (1.1)
c.1199 + 1G > C	p.?	rs62509015	Splice site	Pathogenic	I11	?/?	0	Classic PKU	1 (1.1)
Exon 3 deletion	-	-	Intragenic deletion	Pathogenic	E3	?/?	0	Classic PKU	1 (1.1)

Gene: *PAH*, VUS: ‘Variants of unknown/uncertain significance’ C: catalytic, R: regulatory,?: not known

ENST00000553106.6 and NM_000277.3 transcripts were taken as reference

For interpretation of APV see: Garbade SF, Shen N, Himmelreich N, Haas D, Trefz FK, Hoffmann GF, Burgard P, Blau N (2019) Allelic phenotype values: a model for genotype-based phenotype prediction in phenylketonuria. Genet Med 21(3):580–590. https://doi.org/10.1038/s41436-018-0081-x

APV (Allelic Phenotype Values) = (0: most severe classic PKU; 10: mildest HPA) (0–2.7 is classic PKU; 2.8–6.6 is mild PKU; 6.7–10.0 is mild HPA)

Since one patient’s *PAH* gene analysis was normal (n: 1/48) and the blood Phe levels decreased to < 2 mg/dl during follow-up visits, the patient was diagnosed with transient HPA and was not included in the statistical data. Moreover, two patients were found to have only one allele, for which further genetic analyses are ongoing (n: 2/47). The biochemical profile of one of these patients was included in the mild HPA-not requiring treatment group, while the other was included in the mild PKU group. We did not have any patients in the moderate PKU group who underwent genetic analysis

**Table 2:** Spectrum of observed genotypes in patients with HPA/PKU according to classification proposed by Camp et al. 2014. (n: 47)

Mild HPA-not requiring treatment (*n* = 27)		Mild HPA-gray zone (*n* = 10)		Mild PKU (*n* = 4)		Classic PKU (*n* = 6)	
Genotype	Number of patients/Frequency (%)	Genotype	Number of patients/Frequency (%)	Genotype	Number of patients/Frequency (%)	Genotype	Number of patients/Frequency (%)
c.898G > T p.(Ala300Ser) / c.898G > T p.(Ala300Ser)	3 (11.1)	c.631C > A p.(Pro211Thr) / c.631C > A p.(Pro211Thr)	1 (10)	c.592_613del p.(Tyr198Serfs*136) / c.1169A > G p.(Glu390Gly)	1 (25)	c.842C > T p.(Pro281Leu) / c.842C > T p.(Pro281Leu)	2 (33.3)
c.898G > T p.(Ala300Ser) / c.1066-11G > A p.?	3 (11.1)	c.631C > A p.(Pro211Thr) / c.1066-11G > A p.?	1 (10)	c.631C > A p.(Pro211Thr) / unidentified	1 (25)	c.842C > T p.(Pro281Leu) / c.1066-11G > A p.?	1 (16.7)
c.1208C > T p.(Ala403Val) / c.1208C > T p.(Ala403Val)	2 (7.4)	c.1114A > T p.(Thr372Ser) / c.1114A > T p.(Thr372Ser)	1 (10)	c.722G > A p.(Arg241His) / c.727C > T p.(Arg243*)	1 (25)	c.165delT p.(Phe55Leufs*6) / c.592_613del p.(Tyr198Serfs*136)	1 (16.7)
c.688G > A p.(Val230Ile) / c.688G > A p.(Val230Ile)	1 (3.7)	c.1066-7C > A p.? / c.1066-7C > A p.?	1 (10)	c.898G > T p.(Ala300Ser) / c.1089delG p.(Lys363Asnfs*37)	1 (25)	c.1066-11G > A p.? / c.1066-11G > A p.?	1 (16.7)
c.688G > A p.(Val230Ile) / c.782G > A p.(Arg261Gln)	1 (3.7)	c.533A > G p.(Glu178Gly) / c.673C > A p.(Pro225Thr)	1 (10)			c.1066-7C > A p.? / c.1199 + 1G > C p.?	1 (16.7)
c.631C > A p.(Pro211Thr) / c.631C > A p.(Pro211Thr)	1 (3.7)	c.992 T > C p.(Phe331Ser) / c.1208C > T p.(Ala403Val)	1 (10)				
c.631C > A p.(Pro211Thr) / c.158G > A p.(Arg53His)	1 (3.7)	c.511G > T p.(Gly171Trp) / c.1222C > T p.(Arg408Trp)	1 (10)				
c.355C > T p.(Pro119Ser) / c.355C > T p.(Pro119Ser)	1 (3.7)	c.1089delG p.(Lys363Asnfs*37) / c.1169A > G p.(Glu390Gly)	1 (10)				
c.1139C > T p.(Thr380Met) / c.1139C > T p.(Thr380Met)	1 (3.7)	c.592_613del p.(Tyr198Serfs*136) / c.898G > T p.(Ala300Ser)	1 (10)				
c.533A > G p.(Glu178Gly) / c.533A > G p.(Glu178Gly)	1 (3.7)	c.688G > A p.(Val230Ile) / Exon 3 deletion	1 (10)				
c.722G > A p.(Arg241His) / c.529G > A p.(Val177Met)	1 (3.7)						
c.728G > A p.(Arg243Gln) / c.898G > T p.(Ala300Ser)	1 (3.7)						
c.728G > A p.(Arg243Gln) / c.1208C > T p.(Ala403Val)	1 (3.7)						
c.898G > T p.(Ala300Ser) / c.1208C > T p.(Ala403Val)	1 (3.7)						
c.665A > G p.(Asp222Gly) / c.665A > G p.(Asp222Gly)	1 (3.7)						
c.688G > A p.(Val230Ile) / c.842C > T p.(Pro281Leu)	1 (3.7)						
c.506G > A p.(Arg169His) / c.898G > T p.(Ala300Ser)	1 (3.7)						
c.165 T > G p.(Phe55Leu) / c.1066-11G > A p.?	1 (3.7)						
c.143 T > C p.(Leu48Ser) / c.688G > A p.(Val230Ile)	1 (3.7)						
c.1139C > T p.(Thr380Met) / c.1208C > T p.(Ala403Val)	1 (3.7)						
c.533A > G p.(Glu178Gly) / c.1199 + 5G > T p.?	1 (3.7)						
c.721C > T p.(Arg241Cys) / unidentified	1 (3.7)						

Since one patient’s *PAH* gene analysis was normal (n: 1/48) and the blood Phe levels decreased to < 2 mg/dl during follow-up visits, the patient was diagnosed with transient HPA and was not included in the statistical data. Moreover, two patients were found to have only one allele, for which further genetic analyses are ongoing (n: 2/47). The biochemical profile of one of these patients was included in the mild HPA-not requiring treatment group, while the other was included in the mild PKU group. We did not have any patients in the moderate PKU group who underwent genetic analysis

**Table 3:** Distribution of *PAH* gene variants in the HPA/PKU patients according to classification proposed by Camp et al. 2014. (n: 47)

Mild HPA-not requiring treatment (*n* = 27)		Mild HPA-gray zone (*n* = 10)		Mild PKU (*n* = 4)		Classic PKU (*n* = 6)	
Variant	Number of alleles/allele frequency (%)	Variant	Number of alleles/allele frequency (%)	Variant	Number of alleles/allele frequency (%)	Variant	Number of alleles/allele frequency (%)
c.898G > T p.(Ala300Ser)	12 (22.2)	c.631C > A p.(Pro211Thr)	3 (15)	c.592_613del p.(Tyr198Serfs*136)	1 (12.5)	c.842C > T p.(Pro281Leu)	5 (41.7)
c.1208C > T p.(Ala403Val)	7 (13)	c.1114A > T p.(Thr372Ser)	2 (10)	c.631C > A p.(Pro211Thr)	1 (12.5)	c.1066-11G > A p.?	3 (25)
c.688G > A p.(Val230Ile)	5 (9.3)	c.1066-7C > A p.?	2 (10)	c.722G > A p.(Arg241His)	1 (12.5)	c.165delT p.(Phe55Leufs*6)	1 (8.3)
c.1066-11G > A p.?	4 (7.4)	c.898G > T p.(Ala300Ser)	1 (5)	c.727C > T p.(Arg243*)	1 (12.5)	c.592_613del p.(Tyr198Serfs*136)	1 (8.3)
c.533A > G p.(Glu178Gly)	3 (5.6)	c.1208C > T p.(Ala403Val)	1 (5)	c.898G > T p.(Ala300Ser)	1 (12.5)	c.1066-7C > A p.?	1 (8.3)
c.631C > A p.(Pro211Thr)	3 (5.6)	c.688G > A p.(Val230Ile)	1 (5)	c.1089delG p.(Lys363Asnfs*37)	1 (12.5)	c.1199 + 1G > C p.?	1 (8,3)
c.1139C > T p.(Thr380Met)	3 (5.6)	c.1066-11G > A p.?	1 (5)	c.1169A > G p.(Glu390Gly)	1 (12.5)		
c.355C > T p.(Pro119Ser)	2 (3.7)	c.533A > G p.(Glu178Gly)	1 (5)	Unidentified	1 (12.5)		
c.665A > G p.(Asp222Gly)	2 (3.7)	c.511G > T p.(Gly171Trp)	1 (5)				
c.728G > A p.(Arg243Gln)	2 (3.7)	c.592_613del p.(Tyr198Serfs*136)	1 (5)				
c.143 T > C p.(Leu48Ser)	1 (1.9)	c.673C > A p.(Pro225Thr)	1 (5)				
c.158G > A p.(Arg53His)	1 (1.9)	c.992 T > C p.(Phe331Ser)	1 (5)				
c.165 T > G p.(Phe55Leu)	1 (1.9)	c.1089delG p.(Lys363Asnfs*37)	1 (5)				
c.506G > A p.(Arg169His)	1 (1.9)	c.1169A > G p.(Glu390Gly)	1 (5)				
c.529G > A p.(Val177Met)	1 (1.9)	c.1222C > T p.(Arg408Trp)	1 (5)				
c.721C > T p.(Arg241Cys)	1 (1.9)	Ekzon 3 deletion	1 (5)				
c.722G > A p.(Arg241His)	1 (1.9)						
c.782G > A p.(Arg261Gln)	1 (1.9)						
c.842C > T p.(Pro281Leu)	1 (1.9)						
c.1199 + 5G > T p.?	1 (1.9)						
Unidentified	1 (1.9)						

Since one patient’s *PAH* gene analysis was normal (n: 1/48) and the blood Phe levels decreased to < 2 mg/dl during follow-up visits, the patient was diagnosed with transient HPA and was not included in the statistical data. Moreover, two patients were found to have only one allele, for which further genetic analyses are ongoing (n: 2/47). The biochemical profile of one of these patients was included in the mild HPA-not requiring treatment group, while the other was included in the mild PKU group. We did not have any patients in the moderate PKU group who underwent genetic analysis

**Table 4:** Spectrum of observed *PAH* genotypes in patients with HPA/PKU (n: 47)

Genotype (*n* = 47)	Number of patients/Frequency (%)
c.898G > T p.(Ala300Ser) / c.898G > T p.(Ala300Ser)	3 (6.4)
c.898G > T p.(Ala300Ser) / c.1066-11G > A p.?	3 (6.4)
c.842C > T p.(Pro281Leu) / c.842C > T p.(Pro281Leu)	2 (4.3)
c.1208C > T p.(Ala403Val) / c.1208C > T p.(Ala403Val)	2 (4.3)
c.631C > A p.(Pro211Thr) / c.631C > A p.(Pro211Thr)	2 (4.3)
c.631C > A p.(Pro211Thr) / c.158G > A p.(Arg53His)	1 (2.1)
c.631C > A p.(Pro211Thr) / c.1066-11G > A p.?	1 (2.1)
c.688G > A p.(Val230Ile) / c.688G > A p.(Val230Ile)	1 (2.1)
c.688G > A p.(Val230Ile) / c.782G > A p.(Arg261Gln)	1 (2.1)
c.631C > A p.(Pro211Thr) / unidentified	1 (2.1)
c.355C > T p.(Pro119Ser) / c.355C > T p.(Pro119Ser)	1 (2.1)
c.1066-11G > A p.? / c.1066-11G > A p.?	1 (2.1)
c.1139C > T p.(Thr380Met) / c.1139C > T p.(Thr380Met)	1 (2.1)
c.1114A > T p.(Thr372Ser) / c.1114A > T p.(Thr372Ser)	1 (2.1)
c.533A > G p.(Glu178Gly) / c.533A > G p.(Glu178Gly)	1 (2.1)
c.1066-7C > A p.? /c.1066-7C > A p.?	1 (2.1)
c.722G > A p.(Arg241His) / c.727C > T p.(Arg243*)	1 (2.1)
c.722G > A p.(Arg241His) / c.529G > A p.(Val177Met)	1 (2.1)
c.533A > G p.(Glu178Gly) / c.673C > A p.(Pro225Thr)	1 (2.1)
c.992 T > C p.(Phe331Ser) / c.1208C > T p.(Ala403Val)	1 (2,1)
c.842C > T p.(Pro281Leu) / c.1066-11G > A p.?	1 (2.1)
c.728G > A p.(Arg243Gln) / c.898G > T p.(Ala300Ser)	1 (2.1)
c.728G > A p.(Arg243Gln) / c.1208C > T p.(Ala403Val)	1 (2.1)
c.898G > T p.(Ala300Ser) / c.1208C > T p.(Ala403Val)	1 (2.1)
c.898G > T p.(Ala300Ser) / c.1089delG p.(Lys363Asnfs*37)	1 (2.1)
c.665A > G p.(Asp222Gly) / c.665A > G p.(Asp222Gly)	1 (2.1)
c.511G > T p.(Gly171Trp) / c.1222C > T p.(Arg408Trp)	1 (2.1)
c.1089delG p.(Lys363Asnfs*37) / c.1169A > G p.(Glu390Gly)	1 (2.1)
c.688G > A p.(Val230Ile) / c.842C > T p.(Pro281Leu)	1 (2.1)
c.506G > A p.(Arg169His) / c.898G > T p.(Ala300Ser)	1 (2.1)
c.165 T > G p.(Phe55Leu) / c.1066-11G > A p.?	1 (2.1)
c.165delT p.(Phe55Leufs*6) / c.592_613del p.(Tyr198Serfs*136)	1 (2.1)
c.143 T > C p.(Leu48Ser) / c.688G > A p.(Val230Ile)	1 (2.1)
c.1139C > T p.(Thr380Met) / c.1208C > T p.(Ala403Val)	1 (2.1)
c.592_613del p.(Tyr198Serfs*136) / c.898G > T p.(Ala300Ser)	1 (2.1)
c.592_613del p.(Tyr198Serfs*136) / c.1169A > G p.(Glu390Gly)	1 (2.1)
c.533A > G p.(Glu178Gly) / c.1199 + 5G > T p.?	1 (2.1)
c.1066-7C > A p.? /c.1199 + 1G > C p.?	1 (2.1)
c.688G > A p.(Val230Ile) / Exon 3 deletion	1 (2.1)
c.721C > T p.(Arg241Cys) / unidentified	1 (2.1)

Since one patient’s *PAH* gene analysis was normal (n: 1/48) and the blood Phe levels decreased to < 2 mg/dl during follow-up visits, the patient was diagnosed with transient HPA and was not included in the statistical data. Moreover, two patients were found to have only one allele, for which further genetic analyses are ongoing (n: 2/47). The biochemical profile of one of these patients was included in the mild HPA-not requiring treatment group, while the other was included in the mild PKU group. We did not have any patients in the moderate PKU group who underwent genetic analysis

When grouped by phenotype, the most common variant among mild HPA patients was c.898G > T p.(Ala300Ser) (17.6%), and the most common genotypes were c.898G > T p.(Ala300Ser)/c.898G > T p.(Ala300Ser) (8.1%) and c.898G > T p.(Ala300Ser)/c.1066-11G > A (8.1%). In patients with mild HPA-not requiring treatment, the most common variants were c.898G > T p.(Ala300Ser) (22.2%), c.1208C > T p.(Ala403Val) (13%), c.688G > A p.(Val230Ile) (9.3%), and c.1066-11G > A (7.4%). The three most common genotypes in this group were c.898G > T p.(Ala300Ser)/c.898G > T p.(Ala300Ser) (11.1%), c.898G > T p.(Ala300Ser)/c.1066-11G > A (11.1%), and c.1208C > T p.(Ala403Val)/c.1208C > T p.(Ala403Val) (7.4%). Interestingly, homozygous genotypes such as c.898G > T p.(Ala300Ser)/c.898G > T p.(Ala300Ser) (11.1%), c.1208C > T p.(Ala403Val)/c.1208C > T p.(Ala403Val) (7.4%), c.355C > T p.(Pro119Ser)/c.355C > T p.(Pro119Ser) (3.7%), c.533A > G p.(Glu178Gly)/c.533A > G p.(Glu178Gly) (3.7%), c.665A > G p.(Asp222Gly)/c.665A > G p.(Asp222Gly) (3.7%), c.688G > A p.(Val230Ile)/c.688G > A p.(Val230Ile) (3.7%), and c.1139C > T p.(Thr380Met)/c.1139C > T p.(Thr380Met) (3.7%) were found exclusively in the mild HPA-not requiring treatment group. The three most common variants in mild HPA-gray zone patients were c.631C > A p.(Pro211Thr) (15%), c.1114A > T p.(Thr372Ser) (10%), and c.1066-7C > A (10%). The homozygous genotypes c.1114A > T p.(Thr372Ser)/c.1114A > T p.(Thr372Ser) (10%) and c.1066-7C > A/c.1066-7C > A (10%) were found exclusively in this group. Additionally, the c.631C > A p.(Pro211Thr)/c.631C > A p.(Pro211Thr) homozygous genotype was present in both the mild HPA-not requiring treatment and mild HPA-gray zone groups. When evaluating the allele frequencies in classic PKU patients, the most common variants were c.842C > T p.(Pro281Leu) (41.7%) and c.1066-11G > A (25%). The most frequent genotype in this group was c.842C > T p.(Pro281Leu)/c.842C > T p.(Pro281Leu) (33.3%). Notably, the homozygous genotypes c.842C > T p.(Pro281Leu)/c.842C > T p.(Pro281Leu) (33.3%) and c.1066-11G > A/c.1066-11G > A (16.7%) were observed exclusively in the classic PKU group.

## Discussion

Phenylketonuria is the most common inborn error of amino acid metabolism and can be diagnosed through NS. The disease is primarily managed with a low-Phe diet. While pharmacological treatments, particularly for milder forms of PKU, are available and effective, dietary therapy remains the gold standard for treatment (Esgi et al. 2024).

In our children's hospital, the metabolism unit has been operational for only 9 years, which may explain why the median current age of our patients is relatively low (33 months), as it is still a developing clinic. Although the proportion of female patients (56.6%) was higher than that of male patients (43.4%), no significant gender difference was observed, which is consistent with the autosomal recessive inheritance of PKU. The median age at presentation was 20 days, with most patients presenting during the neonatal period, thanks to effective referrals through NS. To reduce the diagnostic delay and further shorten this period to less than two weeks, more rapid and organized efforts are necessary. However, there were 6 patients who, despite all efforts, missed the screening programs and were diagnosed later. The median age at presentation for this group was 180 days. All of these patients exhibited developmental delays. Five of the patients (two of whom were siblings) were foreign immigrants with a history of first-degree cousin marriages and were diagnosed with classic PKU. The sixth patient also had developmental delay and a history of first-degree cousin marriage; she was diagnosed with PTPS deficiency during follow-up. A moderately significant positive correlation was observed between the age at presentation and blood Phe levels at the time of diagnosis. This may be attributed to the fact that patients who present later are often left untreated initially and may have consumed a less restrictive diet, leading to an increase in blood Phe levels. The relationship between elevated blood Phe levels and cognitive decline has been extensively documented. A meta-analysis suggested that for every 100 μmol/L (approximately 1.66 mg/dL) increase in blood Phe levels, IQ decreases by 1.3 to 3.1 points during childhood (ages 0–12). Additionally, a lifetime increase of 100 μmol/L in Phe levels is associated with an IQ reduction of 1.9 to 4.1 points (Waisbren et al. 2007). This association has been further emphasized in the European guidelines on PKU, which highlight the cognitive impact of elevated Phe levels and underline the importance of early and continuous management of Phe levels (van Wegberg et al. 2017). In 91 (92%) of the patients, the reason for admission to the metabolism unit was early detection through the NS program. The remaining patients presented late due to developmental delay and/or a family history of similar disease. When patients do not participate in the NS program, neurodevelopmental issues due to Phe toxicity become inevitable, as the diagnosis is delayed and the opportunity for early treatment is missed.

The rate of consanguinity among parents in our study was 35.4%, with 20.2% having first-degree cousin marriages, 6.1% having second-degree cousin marriages, and 9.1% having third-degree cousin marriages. This rate is lower compared to previous studies conducted in Turkey, which reported consanguinity rates ranging from 60 to 100% in patients with HPA/PKU (Özgüç et al. 1993). This difference may be explained by two factors: first, the national NS program, which began in Turkey in 2006, has expanded access to early diagnosis, including detection of milder forms of the disease that lack clinical symptoms; second, the high carrier frequency of HPA/PKU (1/25) in Turkey likely contributes to a higher overall number of cases identified through the NS program.

In the present study, similar to recent studies conducted in our country, 75% of the patients had mild HPA (74% of mild HPA-not requiring treatment and 26% mild HPA-gray zone), while 25% had mild, moderate, or severe PKU (Çınar et al. 2022; Erdol and Bilgin 2022; Balasar et al. 2024). These findings support previous reports indicating that classic PKU is more common in Eastern Europe, whereas Mediterranean countries, including Turkey, tend to report milder phenotypes (Hillert et al. 2020). The distribution of metabolic phenotypes appears to correlate with the frequency of regional genotypes. The high rate of mild HPA-not requiring treatment in our study is considered a favorable outcome for both patients and the national healthcare system. Regarding BH4 metabolism disorders, two Turkish patients (2%) were diagnosed, a rate consistent with the literature. The first patient was diagnosed with DHPR deficiency in the early stages, based on low DHPR enzyme activity in the dried blood sample. The second patient, diagnosed with PTPS deficiency, was being investigated for microcephaly, developmental delay, and epilepsy. It was noted that this patient's blood sample had not been collected for NS. This highlights the importance of NS and underscores the need to evaluate all HPA/PKU patients for potential BH4 metabolism disorders.

In this study, dietary therapy was the primary treatment modality. Of the 39 patients (39/99, 39.4%) who received diet therapy, 30 (77%) had good dietary compliance, while 9 (23%) had poor compliance. When evaluating the phenotypes of patients with poor dietary compliance, 4 (44.4%) had classic PKU, 3 (33.3%) had mild HPA-gray zone, 1 (11.1%) had moderate PKU, and 1 (11.1%) had mild PKU. No statistically significant difference in dietary compliance was found between male and female patients. Developmental evaluations of 20 out of 40 patients requiring treatment showed that 13 (65%) had normal development, 2 (10%) had a slightly increased need for support, 1 (5%) had autism (this patient was also diagnosed with Niemann-Pick type C as a coincidental disease), and 4 (20%) had developmental delay. Notably, all patients with developmental delay were diagnosed late and had poor dietary compliance (3 with classic PKU and 1 with PTPS deficiency). Growth parameters were also assessed, revealing that 9 (9.2%) patients had malnutrition, 9 (9.2%) had short stature, and 12 (12.6%) had microcephaly. These growth and developmental delays were most prominently observed in late-diagnosed patients with classic PKU and poor dietary compliance, as well as those with maternal PKU syndrome and BH4 metabolism disorders. This study highlights the critical importance of NS for early diagnosis and treatment. Furthermore, it demonstrates that, as previously reported, multiple metabolic disorders can coincidentally occur in a single patient, such as the coexistence of phenylketonuria and Niemann-Pick Type C in one case (Shickh et al. 2021).

In the present study, disease-causing variants were identified in 49 out of 50 genetically investigated patients with HPA/PKU. The frequency of compound heterozygous genotypes (59.6%) was higher compared to previous studies in our country. This may be due to the gradual decrease in consanguineous marriages and the increased detection of mild HPA-not requiring treatment patients through the national NS program, in conjunction with the high carrier allele frequency (1/25). Consistent with other studies in Turkey, most variants were missense (72.7%). Similar to findings from other studies, exons 6, 7, 8, 11, and intron 10 contained the majority of mutant alleles, further confirming the predominant involvement of the central catalytic domain of the *PAH* gene (Çınar et al. 2022; Erdol and Bilgin 2022; Ozturk and Akin Duman 2022; Balasar et al. 2024).

In our study, the following homozygous alleles were detected: c.898G > T p.(Ala300Ser), c.1208C > T p.(Ala403Val), c.355C > T p.(Pro119Ser), c.533A > G p.(Glu178Gly), c.665A > G p.(Asp222Gly), c.688G > A p.(Val230Ile), and c.1139C > T p.(Thr380Met) in the mild HPA-not requiring treatment group; c.1114A > T p.(Thr372Ser) and c.1066-7C > A in the mild HPA-gray zone group; c.842C > T p.(Pro281Leu) and c.1066-11G > A in the severe PKU group. The homozygous c.631C > A p.(Pro211Thr) allele was found in both the mild HPA-not requiring treatment and mild HPA-gray zone groups. Pathogenic variants associated with a severe phenotype [c.842C > T p.(Pro281Leu), c.1066-11G > A] were found to cause a mild phenotype when combined with variants associated with milder phenotypes [c.898G > T p.(Ala300Ser), c.688G > A p.(Val230Ile), c.165 T > G p.(Phe55Leu), etc.]. This observation aligns with the findings of Garbade et al. (2019), supporting the concept that genotype influences phenotype. In compound heterozygosity, the allele associated with the milder phenotype appears dominant over the allele associated with more severe phenotypes. Since the highest number of patients in our study had mild HPA-not requiring treatment, variants with milder effects formed the highest allele frequencies.

The size of the PAH cohort in this study is relatively modest in comparison to the larger datasets that have been previously published in the literature. Consequently, not all subgroups could be represented. Genetic analyses of some patients in the cohort and cranial imaging or intelligence tests in some patients with intellectual disability were not conducted. Furthermore, BH4 responsiveness was not evaluated due to the limited number of patients receiving sapropterin treatment. Conversely, this cohort is one of the few studies in our country to include genotype–phenotype correlations. The data obtained from this study can be used to generate meta-analysis. Despite HPA/PKU being a common disease in our country, the results of the study indicate that the majority of patient groups exhibit a mild phenotype. This result may prove instructive for determining national health policies.

In conclusion, early diagnosis and prompt treatment of PKU are crucial in preventing intellectual disabilities and other health complications. The mild HPA-not requiring treatment phenotype was the most common in our country. The PAH genotype plays a central role in determining the phenotype of PKU. Newborn screening and early treatment have provided hope for affected individuals. This approach serves as a model for other inborn errors of metabolism that lack screening and treatment programs. We remain deeply grateful to Folling, Bickel, Guthrie, and others for their pioneering work in this field. Their efforts have saved, and will continue to save, thousands of lives worldwide for over 70 years.

## Data Availability

No datasets were generated or analysed during the current study.

## Declarations

This article has not been and will not be published elsewhere in substantially the same form. All co-authors reviewed the final approval of the version from prior to submission. All authors have been involved in (a) conception and design, or analysis and interpretation of data and (b) drafting the case or revising it critically for important intellectual content and agree to submission. All authors have provided the information on ‘Compliance with Ethics Guidelines’.
